# 3-(3-Chloro­phenyl­sulfon­yl)-2,5,7-trimethyl-1-benzofuran

**DOI:** 10.1107/S1600536812008355

**Published:** 2012-03-03

**Authors:** Hong Dae Choi, Pil Ja Seo, Uk Lee

**Affiliations:** a Department of Chemistry, Dongeui University, San 24 Kaya-dong Busanjin-gu, Busan 614-714, Republic of Korea; b Department of Chemistry, Pukyong National University, 599-1 Daeyeon 3-dong, Nam-gu, Busan 608-737, Republic of Korea

## Abstract

In the title compound, C_17_H_15_ClO_3_S, the 3-chloro­phenyl ring makes a dihedral angle of 77.76 (6)° with the mean plane [r.m.s. deviation = 0.007 (1) Å] of the benzofuran fragment. In the crystal, mol­ecules are linked by weak inter­molecular C—H⋯O and C—H⋯π inter­actions.

## Related literature

For background information and the crystal structures of related compounds, see: Choi *et al.* (2008 ▶, 2010 ▶); Seo *et al.* (2011 ▶).
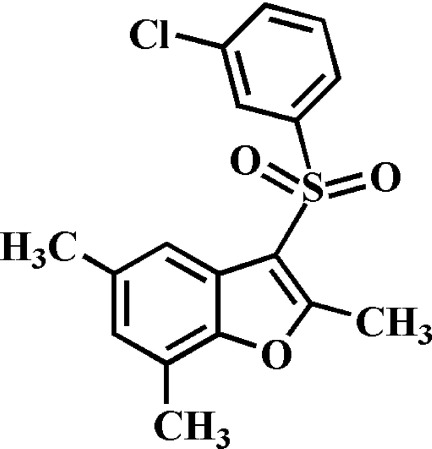


## Experimental

### 

#### Crystal data

C_17_H_15_ClO_3_S
*M* *_r_* = 334.80
Monoclinic, 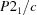

*a* = 14.5299 (3) Å
*b* = 12.9778 (2) Å
*c* = 8.1776 (1) Å
β = 92.615 (1)°
*V* = 1540.41 (4) Å^3^
*Z* = 4
Mo *K*α radiation
μ = 0.39 mm^−1^
*T* = 173 K
0.36 × 0.29 × 0.25 mm

#### Data collection

Bruker SMART APEXII CCD diffractometer
Absorption correction: multi-scan (*SADABS*; Bruker, 2009 ▶) *T*_min_ = 0.872, *T*_max_ = 0.908
14420 measured reflections
3551 independent reflections
3053 reflections with *I* > 2σ(*I*)
*R*_int_ = 0.028

#### Refinement

*R*[*F* ^2^ > 2σ(*F* ^2^)] = 0.038
*wR*(*F* ^2^) = 0.105
*S* = 1.04
3551 reflections
202 parameters
H-atom parameters constrained
Δρ_max_ = 0.31 e Å^−3^
Δρ_min_ = −0.41 e Å^−3^

### 

Data collection: *APEX2* (Bruker, 2009 ▶); cell refinement: *SAINT* (Bruker, 2009 ▶); data reduction: *SAINT*; program(s) used to solve structure: *SHELXS97* (Sheldrick, 2008 ▶); program(s) used to refine structure: *SHELXL97* (Sheldrick, 2008 ▶); molecular graphics: *ORTEP-3* (Farrugia, 1997 ▶) and *DIAMOND* (Brandenburg, 1998 ▶); software used to prepare material for publication: *SHELXL97*.

## Supplementary Material

Crystal structure: contains datablock(s) global, I. DOI: 10.1107/S1600536812008355/bh2417sup1.cif

Structure factors: contains datablock(s) I. DOI: 10.1107/S1600536812008355/bh2417Isup2.hkl

Supplementary material file. DOI: 10.1107/S1600536812008355/bh2417Isup3.cml

Additional supplementary materials:  crystallographic information; 3D view; checkCIF report

## Figures and Tables

**Table 1:** Hydrogen-bond geometry (Å, °) *Cg*1 and *Cg*2 are the centroids of the C1/C2/C7/O1/C8 furan ring and the C12–C17 benzene ring, respectively.

*D*—H⋯*A*	*D*—H	H⋯*A*	*D*⋯*A*	*D*—H⋯*A*
C14—H14⋯O3^i^	0.95	2.60	3.264 (2)	127
C10—H10*A*⋯*Cg*2^ii^	0.98	2.86	3.704 (2)	145
C10—H10*C*⋯*Cg*1^ii^	0.98	3.08	3.536 (2)	110

Symmetry codes: (i) 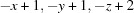; (ii) 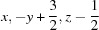.

**Fig. 1.**
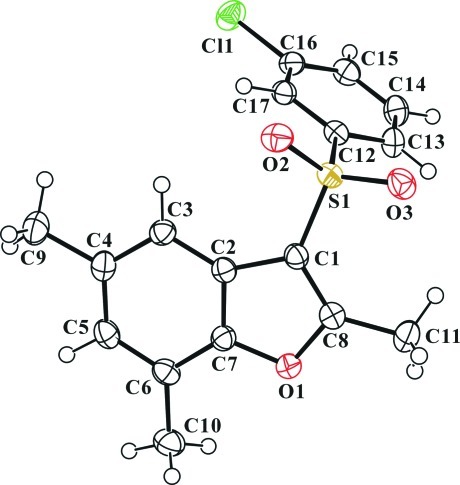
The molecular structure of the title compound with displacement ellipsoids drawn at the 50% probability level. H atoms are presented as small spheres of arbitrary radius.

**Fig. 2.**
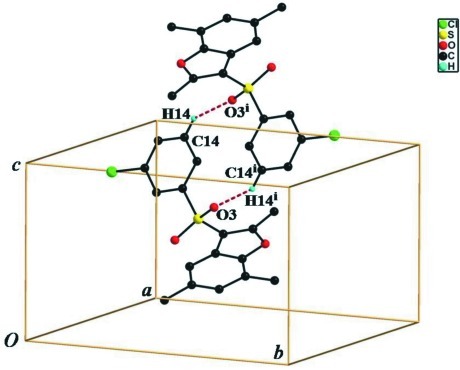
A view of the C–H···O interactions (dotted lines) in the crystal structure of the title compound. H atoms non-participating in hydrogen-bonding were omitted for clarity. Symmetry code: (i) -x+1, -y+1, -z+2].

**Fig. 3.**
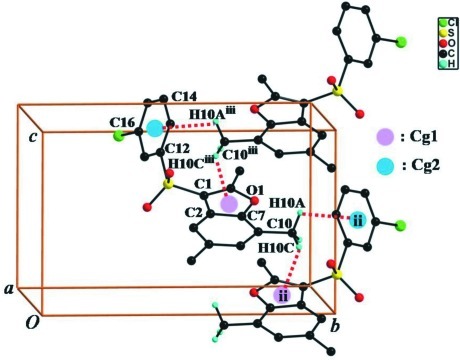
A view of the C–H···π interactions (dotted lines) in the crystal structure of the title compound. H atoms non-participating in hydrogen-bonding were omitted for clarity [Symmetry codes: (ii) x, -y +3/2, z-1/2; (iii) x, -y+3/2, z+1/2].

## supplementary crystallographic information

### Comment

As a part of our ongoing study of 2,5,7-trimethyl-1-benzofuran derivatives containing 3-phenylsulfonyl (Choi *et al.*, 2008), 3-(4-fluorophenylsulfonyl) (Choi *et al.*, 2010) and 3-(3-fluorophenylsulfonyl) (Seo *et al.*, 2011) substituents, we report herein the crystal structure of the title compound.

In the title molecule (Fig. 1), the benzofuran unit is essentially planar, with a mean deviation of 0.007 (1) Å from the least-squares plane defined by the nine constituent atoms. The dihedral angle between the 3-chlorophenyl ring and the mean plane of the benzofuran fragment is 77.76 (6)°. The crystal packing is stabilized by weak intermolecular C–H···O hydrogen bonds (Fig. 2 & Table 1). The crystal packing is further stabilized by intermolecular C–H···π interactions (Fig.3 & Table 1, Cg1 and Cg2 are the centroids of the C1/C2/ C7/O1/C8 furan ring and the C12-C17 benzene ring, respectively).

### Experimental

77% 3-Chloroperoxybenzoic acid (493 mg, 2.2 mmol) was added in small portions to a stirred solution of 3-(3-chlorophenylsulfanyl)-2,5,7-trimethyl-1-benzofuran (333 mg, 1.1 mmol) in dichloromethane (40 mL) at 273 K. After being stirred at room temperature for 10 h., the mixture was washed with saturated sodium bicarbonate solution and the organic layer was separated, dried over magnesium sulfate, filtered and concentrated at reduced pressure. The residue was purified by column chromatography (benzene) to afford the title compound as a colorless solid [yield 71%, m.p. 413-414 K; *R*_f_= 0.46 (benzene)]. Single crystals suitable for X-ray diffraction were prepared by slow evaporation of a solution of the title compound in acetone, at room temperature.

### Refinement

All H atoms were positioned geometrically and refined using a riding model, with C–H = 0.95 Å for aryl and 0.98 Å for methyl H atoms. *U*_iso_(H) =1.2*U*_eq_(C) for aryl and 1.5*U*_eq_(C)for methyl H atoms.

### Crystal data

**Table:**

C_17_H_15_ClO_3_S	*F*(000) = 696
*M**_r_* = 334.80	*D*_x_ = 1.444 Mg m^−^^3^
Monoclinic, *P*2_1_/*c*	Melting point: 413 K
Hall symbol: -P 2ybc	Mo *K*α radiation, λ = 0.71073 Å
*a* = 14.5299 (3) Å	Cell parameters from 7514 reflections
*b* = 12.9778 (2) Å	θ = 2.8–27.4°
*c* = 8.1776 (1) Å	µ = 0.39 mm^−^^1^
β = 92.615 (1)°	*T* = 173 K
*V* = 1540.41 (4) Å^3^	Block, colourless
*Z* = 4	0.36 × 0.29 × 0.25 mm

### Data collection

**Table:**

Bruker SMART APEXII CCD diffractometer	3551 independent reflections
Radiation source: rotating anode	3053 reflections with *I* > 2σ(*I*)
Graphite multilayer monochromator	*R*_int_ = 0.028
Detector resolution: 10.0 pixels mm^-1^	θ_max_ = 27.5°, θ_min_ = 1.4°
φ and ω scans	*h* = −15→18
Absorption correction: multi-scan (*SADABS*; Bruker, 2009)	*k* = −15→16
*T*_min_ = 0.872, *T*_max_ = 0.908	*l* = −10→10
14420 measured reflections	

### Refinement

**Table:**

Refinement on *F*^2^	Primary atom site location: structure-invariant direct methods
Least-squares matrix: full	Secondary atom site location: difference Fourier map
*R*[*F*^2^ > 2σ(*F*^2^)] = 0.038	Hydrogen site location: difference Fourier map
*wR*(*F*^2^) = 0.105	H-atom parameters constrained
*S* = 1.04	*w* = 1/[σ^2^(*F*_o_^2^) + (0.0542*P*)^2^ + 0.775*P*] where *P* = (*F*_o_^2^ + 2*F*_c_^2^)/3
3551 reflections	(Δ/σ)_max_ < 0.001
202 parameters	Δρ_max_ = 0.31 e Å^−^^3^
0 restraints	Δρ_min_ = −0.41 e Å^−^^3^
0 constraints	

### Fractional atomic coordinates and isotropic or equivalent isotropic displacement parameters (Å^2^)

**Table:**

	*x*	*y*	*z*	*U*_iso_*/*U*_eq_	
Cl1	0.11297 (3)	0.26899 (4)	0.95927 (6)	0.03925 (14)	
S1	0.38836 (3)	0.46155 (3)	0.65400 (5)	0.02434 (12)	
O1	0.32039 (8)	0.75011 (9)	0.56955 (15)	0.0285 (3)	
O2	0.35944 (9)	0.38758 (10)	0.53201 (14)	0.0313 (3)	
O3	0.48438 (8)	0.47396 (10)	0.69791 (15)	0.0325 (3)	
C1	0.34200 (11)	0.57998 (13)	0.59670 (19)	0.0242 (3)	
C2	0.25351 (11)	0.59471 (13)	0.51051 (18)	0.0234 (3)	
C3	0.18368 (12)	0.53119 (13)	0.4455 (2)	0.0265 (4)	
H3	0.1880	0.4584	0.4548	0.032*	
C4	0.10747 (12)	0.57732 (15)	0.3666 (2)	0.0297 (4)	
C5	0.10147 (12)	0.68466 (15)	0.3578 (2)	0.0305 (4)	
H5	0.0485	0.7142	0.3040	0.037*	
C6	0.16886 (12)	0.75061 (14)	0.4236 (2)	0.0281 (4)	
C7	0.24429 (11)	0.70102 (13)	0.49726 (19)	0.0252 (3)	
C8	0.37838 (12)	0.67465 (14)	0.6292 (2)	0.0278 (4)	
C9	0.03198 (14)	0.51129 (17)	0.2890 (3)	0.0404 (5)	
H9A	0.0450	0.4981	0.1743	0.061*	
H9B	−0.0271	0.5472	0.2943	0.061*	
H9C	0.0291	0.4457	0.3479	0.061*	
C10	0.16093 (14)	0.86578 (15)	0.4170 (3)	0.0381 (4)	
H10A	0.1800	0.8948	0.5239	0.057*	
H10B	0.0969	0.8851	0.3895	0.057*	
H10C	0.2007	0.8927	0.3334	0.057*	
C11	0.46509 (13)	0.71222 (16)	0.7113 (3)	0.0385 (4)	
H11A	0.4854	0.6634	0.7970	0.058*	
H11B	0.4544	0.7798	0.7606	0.058*	
H11C	0.5127	0.7183	0.6308	0.058*	
C12	0.33205 (11)	0.42967 (13)	0.83512 (19)	0.0241 (3)	
C13	0.36775 (12)	0.46631 (15)	0.9846 (2)	0.0300 (4)	
H13	0.4209	0.5090	0.9896	0.036*	
C14	0.32471 (13)	0.43965 (16)	1.1264 (2)	0.0335 (4)	
H14	0.3488	0.4634	1.2296	0.040*	
C15	0.24674 (13)	0.37856 (14)	1.1179 (2)	0.0308 (4)	
H15	0.2174	0.3597	1.2150	0.037*	
C16	0.21193 (12)	0.34517 (13)	0.9675 (2)	0.0273 (4)	
C17	0.25354 (11)	0.36921 (13)	0.8236 (2)	0.0253 (3)	
H17	0.2292	0.3452	0.7208	0.030*	

### Atomic displacement parameters (Å^2^)

**Table:**

	*U*^11^	*U*^22^	*U*^33^	*U*^12^	*U*^13^	*U*^23^
Cl1	0.0390 (3)	0.0409 (3)	0.0380 (3)	−0.0132 (2)	0.0041 (2)	0.0024 (2)
S1	0.0259 (2)	0.0245 (2)	0.0226 (2)	0.00192 (15)	0.00121 (15)	0.00141 (15)
O1	0.0278 (6)	0.0236 (6)	0.0338 (7)	−0.0011 (5)	−0.0036 (5)	0.0008 (5)
O2	0.0416 (7)	0.0275 (7)	0.0249 (6)	0.0006 (5)	0.0037 (5)	−0.0028 (5)
O3	0.0252 (6)	0.0372 (7)	0.0352 (7)	0.0032 (5)	0.0020 (5)	0.0056 (6)
C1	0.0245 (8)	0.0261 (9)	0.0219 (7)	0.0003 (6)	−0.0005 (6)	0.0014 (6)
C2	0.0245 (8)	0.0262 (8)	0.0196 (7)	0.0003 (6)	0.0021 (6)	0.0008 (6)
C3	0.0291 (8)	0.0255 (9)	0.0249 (8)	−0.0021 (7)	0.0016 (6)	−0.0008 (7)
C4	0.0263 (8)	0.0377 (10)	0.0251 (8)	−0.0039 (7)	−0.0003 (7)	−0.0010 (7)
C5	0.0242 (8)	0.0373 (10)	0.0298 (8)	0.0037 (7)	−0.0003 (7)	0.0040 (7)
C6	0.0278 (8)	0.0295 (9)	0.0273 (8)	0.0036 (7)	0.0040 (7)	0.0039 (7)
C7	0.0262 (8)	0.0260 (9)	0.0233 (8)	−0.0019 (6)	0.0006 (6)	0.0002 (7)
C8	0.0275 (8)	0.0283 (9)	0.0273 (8)	0.0005 (7)	−0.0008 (7)	0.0011 (7)
C9	0.0328 (10)	0.0460 (12)	0.0417 (11)	−0.0079 (9)	−0.0073 (8)	−0.0002 (9)
C10	0.0379 (10)	0.0305 (10)	0.0456 (11)	0.0052 (8)	0.0001 (8)	0.0059 (8)
C11	0.0329 (9)	0.0353 (11)	0.0463 (11)	−0.0057 (8)	−0.0092 (8)	−0.0038 (9)
C12	0.0254 (8)	0.0240 (8)	0.0227 (7)	0.0033 (6)	0.0005 (6)	0.0014 (6)
C13	0.0258 (8)	0.0373 (10)	0.0263 (8)	−0.0014 (7)	−0.0033 (7)	−0.0013 (7)
C14	0.0361 (10)	0.0419 (11)	0.0220 (8)	0.0005 (8)	−0.0038 (7)	−0.0032 (7)
C15	0.0360 (9)	0.0336 (10)	0.0229 (8)	0.0031 (7)	0.0035 (7)	0.0021 (7)
C16	0.0285 (8)	0.0235 (8)	0.0298 (8)	−0.0006 (7)	0.0013 (7)	0.0018 (7)
C17	0.0305 (8)	0.0230 (8)	0.0221 (7)	0.0010 (7)	−0.0019 (6)	−0.0007 (6)

### Geometric parameters (Å, º)

**Table:**

Cl1—C16	1.7437 (18)	C9—H9A	0.9800
S1—O2	1.4336 (13)	C9—H9B	0.9800
S1—O3	1.4337 (13)	C9—H9C	0.9800
S1—C1	1.7341 (17)	C10—H10A	0.9800
S1—C12	1.7734 (16)	C10—H10B	0.9800
O1—C8	1.367 (2)	C10—H10C	0.9800
O1—C7	1.385 (2)	C11—H11A	0.9800
C1—C8	1.359 (2)	C11—H11B	0.9800
C1—C2	1.450 (2)	C11—H11C	0.9800
C2—C7	1.390 (2)	C12—C17	1.384 (2)
C2—C3	1.394 (2)	C12—C13	1.390 (2)
C3—C4	1.391 (2)	C13—C14	1.386 (2)
C3—H3	0.9500	C13—H13	0.9500
C4—C5	1.397 (3)	C14—C15	1.382 (3)
C4—C9	1.509 (3)	C14—H14	0.9500
C5—C6	1.390 (3)	C15—C16	1.378 (2)
C5—H5	0.9500	C15—H15	0.9500
C6—C7	1.385 (2)	C16—C17	1.383 (2)
C6—C10	1.500 (3)	C17—H17	0.9500
C8—C11	1.483 (2)		
			
O2—S1—O3	120.04 (8)	C4—C9—H9C	109.5
O2—S1—C1	107.91 (8)	H9A—C9—H9C	109.5
O3—S1—C1	109.25 (8)	H9B—C9—H9C	109.5
O2—S1—C12	107.05 (8)	C6—C10—H10A	109.5
O3—S1—C12	107.32 (8)	C6—C10—H10B	109.5
C1—S1—C12	104.13 (8)	H10A—C10—H10B	109.5
C8—O1—C7	106.84 (13)	C6—C10—H10C	109.5
C8—C1—C2	107.71 (15)	H10A—C10—H10C	109.5
C8—C1—S1	127.28 (13)	H10B—C10—H10C	109.5
C2—C1—S1	124.96 (13)	C8—C11—H11A	109.5
C7—C2—C3	119.44 (15)	C8—C11—H11B	109.5
C7—C2—C1	104.41 (15)	H11A—C11—H11B	109.5
C3—C2—C1	136.15 (16)	C8—C11—H11C	109.5
C4—C3—C2	118.20 (16)	H11A—C11—H11C	109.5
C4—C3—H3	120.9	H11B—C11—H11C	109.5
C2—C3—H3	120.9	C17—C12—C13	121.80 (15)
C3—C4—C5	119.95 (16)	C17—C12—S1	119.06 (12)
C3—C4—C9	119.90 (17)	C13—C12—S1	119.14 (13)
C5—C4—C9	120.15 (17)	C14—C13—C12	119.07 (16)
C6—C5—C4	123.56 (16)	C14—C13—H13	120.5
C6—C5—H5	118.2	C12—C13—H13	120.5
C4—C5—H5	118.2	C15—C14—C13	120.09 (16)
C7—C6—C5	114.31 (16)	C15—C14—H14	120.0
C7—C6—C10	122.45 (17)	C13—C14—H14	120.0
C5—C6—C10	123.24 (17)	C16—C15—C14	119.46 (16)
C6—C7—O1	124.92 (16)	C16—C15—H15	120.3
C6—C7—C2	124.52 (16)	C14—C15—H15	120.3
O1—C7—C2	110.55 (14)	C15—C16—C17	122.10 (16)
C1—C8—O1	110.49 (14)	C15—C16—Cl1	118.76 (13)
C1—C8—C11	134.48 (17)	C17—C16—Cl1	119.14 (13)
O1—C8—C11	115.03 (15)	C16—C17—C12	117.46 (15)
C4—C9—H9A	109.5	C16—C17—H17	121.3
C4—C9—H9B	109.5	C12—C17—H17	121.3
H9A—C9—H9B	109.5		
			
O2—S1—C1—C8	−149.77 (15)	C1—C2—C7—C6	−179.53 (15)
O3—S1—C1—C8	−17.69 (18)	C3—C2—C7—O1	179.81 (14)
C12—S1—C1—C8	96.71 (17)	C1—C2—C7—O1	−0.22 (18)
O2—S1—C1—C2	33.36 (16)	C2—C1—C8—O1	−0.38 (19)
O3—S1—C1—C2	165.44 (13)	S1—C1—C8—O1	−177.69 (12)
C12—S1—C1—C2	−80.16 (15)	C2—C1—C8—C11	−179.72 (19)
C8—C1—C2—C7	0.36 (18)	S1—C1—C8—C11	3.0 (3)
S1—C1—C2—C7	177.75 (12)	C7—O1—C8—C1	0.25 (18)
C8—C1—C2—C3	−179.68 (18)	C7—O1—C8—C11	179.72 (15)
S1—C1—C2—C3	−2.3 (3)	O2—S1—C12—C17	−20.30 (15)
C7—C2—C3—C4	1.1 (2)	O3—S1—C12—C17	−150.40 (13)
C1—C2—C3—C4	−178.86 (17)	C1—S1—C12—C17	93.83 (14)
C2—C3—C4—C5	−1.6 (2)	O2—S1—C12—C13	159.90 (14)
C2—C3—C4—C9	177.74 (16)	O3—S1—C12—C13	29.80 (16)
C3—C4—C5—C6	0.5 (3)	C1—S1—C12—C13	−85.96 (15)
C9—C4—C5—C6	−178.82 (17)	C17—C12—C13—C14	1.5 (3)
C4—C5—C6—C7	1.0 (3)	S1—C12—C13—C14	−178.74 (14)
C4—C5—C6—C10	−178.55 (18)	C12—C13—C14—C15	−0.8 (3)
C5—C6—C7—O1	179.26 (15)	C13—C14—C15—C16	−0.5 (3)
C10—C6—C7—O1	−1.2 (3)	C14—C15—C16—C17	1.2 (3)
C5—C6—C7—C2	−1.5 (2)	C14—C15—C16—Cl1	−179.50 (15)
C10—C6—C7—C2	178.05 (17)	C15—C16—C17—C12	−0.6 (3)
C8—O1—C7—C6	179.31 (16)	Cl1—C16—C17—C12	−179.88 (13)
C8—O1—C7—C2	0.00 (18)	C13—C12—C17—C16	−0.8 (2)
C3—C2—C7—C6	0.5 (3)	S1—C12—C17—C16	179.45 (12)

### Hydrogen-bond geometry (Å, º)

*Cg*1 and *Cg*2 are the centroids of the C1/C2/C7/O1/C8 furan ring and the C12-C17 benzene ring, respectively.

**Table:**

*D*—H···*A*	*D*—H	H···*A*	*D*···*A*	*D*—H···*A*
C14—H14···O3^i^	0.95	2.60	3.264 (2)	127
C10—H10*A*···*Cg*2^ii^	0.98	2.86	3.704 (2)	145
C10—H10*C*···*Cg*1^ii^	0.98	3.08	3.536 (2)	110

Symmetry codes: (i) −*x*+1, −*y*+1, −*z*+2; (ii) *x*, −*y*+3/2, *z*−1/2.
